# Longitudinal Dynamics of Cellular Responses in Recovered COVID-19 Patients

**DOI:** 10.3389/fimmu.2022.911859

**Published:** 2022-05-19

**Authors:** Meng-Li Cheng, Hui-Ying Liu, Chao Zhou, Rui-Ting Li, Jing Zheng, Yan-Hong Qin, Ning Yang, Yue Zhang, Juan-Juan Huang, Zhu Zhu, Qing-Yu Meng, Guo-Qing Wang, Hui Zhao, Yun Chen, Chang-Qing Bai, Cheng-Feng Qin, Fan Li

**Affiliations:** ^1^ Department of Pathogenobiology, The Key Laboratory of Zoonosis Research, Chinese Ministry of Education, College of Basic Medical Science, Jilin University, Changchun, China; ^2^ Department of Respiratory and Critical Care Diseases, The Fifth Medical Center, Chinese People's Liberation Army (PLA) General Hospital, Beijing, China; ^3^ Department of Virology, State Key Laboratory of Pathogen and Biosecurity, Beijing Institute of Microbiology and Epidemiology, Academy of Military Medical Sciences, Beijing, China; ^4^ Department of Immunology, Key Laboratory of Immune Microenvironment and Disease, Nanjing Medical University, Nanjing, China; ^5^ Department of Respiratory and Critical Care Medicine, Shenzhen University General Hospital, Shenzhen University, Shenzhen, China; ^6^ China-Japan Union Hospital, Jilin University, Changchun, China

**Keywords:** COVID-19, cellular immunity, Th1 cytokines, interferon, SARS-CoV-2

## Abstract

Safe and effective vaccines and therapeutics based on the understanding of antiviral immunity are urgently needed to end the COVID-19 pandemic. However, the understanding of these immune responses, especially cellular immune responses to SARS-CoV-2 infection, is limited. Here, we conducted a cohort study of COVID-19 patients who were followed and had blood collected to characterize the longitudinal dynamics of their cellular immune responses. Compared with healthy controls, the percentage of activation of SARS-CoV-2 S/N-specific T cells in recovered patients was significantly higher. And the activation percentage of S/N-specific CD8^+^ T cells in recovered patients was significantly higher than that of CD4^+^ T cells. Notably, SARS-CoV-2 specific T-cell responses were strongly biased toward the expression of Th1 cytokines, included the cytokines IFNγ, TNFα and IL2. Moreover, the secreted IFNγ and IL2 level in severe patients was higher than that in mild patients. Additionally, the number of IFNγ-secreting S-specific T cells in recovered patients were higher than that of N-specific T cells. Overall, the SARS-CoV-2 S/N-specific T-cell responses in recovered patients were strong, and virus-specific immunity was present until 14-16 weeks after symptom onset. Our work provides a basis for understanding the immune responses and pathogenesis of COVID-19. It also has implications for vaccine development and optimization and speeding up the licensing of the next generation of COVID-19 vaccines.

## Introduction

Coronavirus disease 2019 (COVID-19) (1, 2) is caused by severe acute respiratory syndrome coronavirus 2 (SARS-CoV-2), which is closely related to SARS-CoV (2–4), and quickly spread throughout the world to cause global public health crises, being declared a pandemic by the World Health Organization. The clinical manifestations of COVID-19 range from nonsymptomatic infection and mild flu-like symptoms to pneumonia, severe acute respiratory distress syndrome and even death (5, 6). As of 10 March 2022, COVID-19 had resulted in more than 451 million confirmed cases and 6.0 million mortalities in the 26 months since it was first identified in December 2019 (7). Encouragingly, the scientific advances related to understanding SARS-CoV-2 and COVID-19 have been extraordinarily rapid and broad. However, the immunology of COVID-19, especially the associated cellular immunity, is poorly understood.

Humans make SARS-CoV-2-specific antibodies, CD4^+^ T cells, and CD8^+^ T cells in response to SARS-CoV-2 infection (8–10). While much attention has been placed on antibody-based immunity, there is increasing evidence that T cells play vital roles in the resolution of COVID-19 (11). T-cell responses have been detected after almost all SARS-CoV-2 infections and in recovered patients (8, 12–14). Studies of acute and convalescent COVID-19 patients have found that SARS-CoV-2-specific T-cell responses are significantly associated with milder disease (13–16), suggesting that T-cell responses may be important for the control and resolution of primary SARS-CoV-2 infection. Additionally, it is critically important to understand whether functional immune memory cells form in recovered COVID-19 patients to reduce transmission and disease. Although memory T-cell responses have been detected in recovered COVID-19 patients, the dynamics are still unclear.

Recently, many SARS-CoV-2 variants have been detected in different regions, and the most paramount variants of concern (VOCs) have attracted global attention (17). These VOCs can enhance the interactions with the host receptor ACE2, increase viral transmissibility or reduce the potency of neutralizing antibodies, thereby compromising the immune responses in recovered COVID-19 patients or efficacy in vaccinated individuals; these features have resulted in increasing breakthrough infections by VOCs both in both recovered COVID-19 patients and vaccinated individuals. Interestingly, although neutralizing activity against VOCs is reduced (17–19), there is no significant difference in memory T-cell responses between the original strain and VOCs (20, 21). Thus, there is an urgent need to better understand the mechanisms of protection mediated by cellular immunity in COVID-19 patients, which may be used to guide vaccine development and optimization and speed up the licensing of the next generation of COVID-19 vaccines.

Therefore, we conducted a cohort study including 23 hospitalized confirmed COVID-19 patients who were followed and had their blood collected. Previously, we evaluated the longitudinal dynamics of humoral immunity in the study cohort, and our results indicated that high-affinity and high-efficiency neutralizing antibodies could last for at least 10 months (22). In this study, we characterized the longitudinal dynamics of cellular immunity in recovered COVID-19 patients.

## Materials And Methods

### Human Samples and Study Design

Between Mar 16, 2020 and May 19, 2020, 23 recovered patients infected with the Wuhan-Hu-1 strain were enrolled, including 12 mild patients and 11 severe patients (**Supplementary Table S1**), as well as 10 healthy donors. The COVID-19 case definition and clinical classification based on severity were defined according to the New Coronavirus Pneumonia Prevention and Control Protocol for COVID-19 (6th edition) released by the National Health Commission of China. To study the dynamics of antibody responses, blood samples were collected successively. Among the 23 recovered patients, 17, including 6 in the mild group and 11 in the severe group, were followed from 8 to 16 weeks after symptom onset. The 23 recovered patients were selected to study longitudinal changes in cellular immune responses and were tested 1 to 3 times. Including the samples from the 6 recovered patients with only 1 blood sample collected, a total of 45 blood samples were analyzed in this study (**Supplementary Table S1**).

Demographic data, clinical manifestations, pathological characteristics, and laboratory findings were collected. Cellular immunity was detected by enzyme-linked immunosorbent (ELISpot) assay, including analysis of IFNγ, TNFα, interleukin (IL) 2, IL4 and IL6, and by flow cytometry, including analysis of different immune cell subsets, macrophages, T cell subsets, and the cytokines IFNγ and TNFα. Due to an insufficient number of peripheral blood mononuclear cells (PBMCs), we chose to detect IFNγ, the most representative cytokine for cellular immunity, during weeks 11-13.

### Serum and Isolation of PBMCs

Serum samples were heated-inactivated (56°C for 1 h) and then stored at -80°C. PBMCs were isolated from anticoagulant-treated blood by density-gradient centrifugation using Ficoll-Hypaque gradients (GE Healthcare Life Sciences, Philadelphia, PA) according to the manufacturer’s instructions. Isolated PBMCs were cryopreserved and stored in liquid nitrogen until used in assays.

### ELISpot Assay

The cellular immune responses in recovered COVID-19 patients were measured using Human IFNγ, TNFα, IL2, IL4, or IL6 precoated ELISpotPRO kits (MabTech), according to the manufacturer’s instructions. In brief, PBMCs were seeded in duplicate in 96-well plates (2x10^5^ cells/well) and stimulated for 48 h with pools of SARS-CoV-2 S protein peptides or N protein peptides (2 μg/ml per peptide, see **Supplementary Table S2**). The T cell peptides used in this study were identified by sequence homology and by *a priori* epitope prediction using bioinformatics approaches to identify potential targets for immune responses to the SARS-CoV-2 (23), and it has been widely recognized (24–26). An anti-CD3 monoclonal antibody (mAb) was used as a positive control, and RPMI 1640 medium was used as a negative control. Spot-forming units (SFU) were quantified with an ImmunoSpot analyzer and counted using an AID ELISpot Reader (vSpot Spectrum). The results are expressed as SFU per 10^6^ cells; a sample was considered positive if it produced 50 or more spots and was at least twice the negative control.

### FACS Staining

For phenotypic analysis, cells were incubated for 20 min at room temperature with anti-CD4 (FITC) (BioLegend), anti-CD3 (PerCP-Cy5.5) (BioLegend), anti-CD8 (PE-Cy7) (BD), anti-CD56 (PE) (BioLegend), anti-CD19 (APC) (BioLegend) and live/dead fixable aqua dye (APC-Cy7) (eBioscience). For macrophage analysis, cells were incubated for 20 min at room temperature with anti-CD86 (PE) (BioLegend), anti-CD68 (PE-Cy7) (BioLegend), anti-CD163 (PerCP-Cy5.5) (BD), and live/dead fixable aqua dye (APC-Cy7) (eBioscience); washed with PBS; fixed; permeabilized; and stained with anti-CD14 (APC) (BioLegend) and anti-CD206 (BB515) (BioLegend). For T cell subsets analysis, cells were incubated for 20 min at room temperature with anti-CD4 (FITC) (BioLegend), anti-CD3 (PE-Cy7) (BioLegend), anti-CD8 (PerCP-Cy5.5) (BD), anti-CCR7 (PE) (BioLegend), anti-CD45RA (APC) (BioLegend) and live/dead fixable aqua dye (APC-Cy7) (eBioscience). Data were analyzed using FlowJo software (version 10.0.8, Tree Star Inc.). For the analysis of phenotypic, macrophage or T cell subsets, an unpaired non-parametric Student’s t test of two independent-samples was used to determine the statistical significance between mild/severe patients and healthy controls.

For SARS-CoV-2-specific T-cell analysis, cells were cultured in 1640 medium supplemented with 10% fetal bovine serum (FBS) and then stimulated with the SARS-CoV-2 S or N protein peptide pool, DMSO (negative control) or PMA (positive control), respectively, and anti-CD28 and anti-CD49d were added as co-stimulation. One hour later, brefeldin A was added. After 16 h of stimulation, the cells were divided into two parts. For T-cell activation analysis, cells were incubated for 20 min at room temperature with anti-CD4 (FITC) (BioLegend), anti-CD3 (PerCP-Cy5.5) (BioLegend), anti-CD8 (PE-Cy7) (BD), anti-CD38 (PE) (BioLegend), and live/dead fixable aqua dye (APC-Cy7) (eBioscience). The other parts were used for T-cell response analysis. In this analysis, cells were incubated for 20 min at room temperature with anti-CD4 (FITC) (BioLegend), anti-CD3 (PE-Cy7) (BioLegend), anti-CD8 (PerCP-Cy5.5) (BD), and live/dead fixable aqua dye (APC-Cy7) (eBioscience); washed with PBS; fixed; permeabilized; and stained with anti-IFNγ (APC) (BioLegend) and anti-TNFα (PE) (BioLegend). After staining, cells were washed with PBS and stored at 4°C until acquired on a FACSVerse (BD Biosciences, San Jose, CA). Data were analyzed using FlowJo software (version 10.0.8, Tree Star Inc.). For the analysis of SARS-CoV-2-specific T cells, the percentage of DMSO-stimulated PBMCs was subtracted from that of the peptide-stimulated PBMCs, if the percentage of SARS-CoV-2-specific T cells greater than zero are considered positive, and those equal to zero are negative. An unpaired non-parametric Student’s t test of two independent-samples was used to determine the statistical significance between mild/severe patients and healthy controls.

### Statistical Analysis

All data were analyzed with GraphPad Prism 8.0 software. Data are presented as the mean ± SEM in all experiments or as described in the corresponding legends. One-way ANOVA analysis of variance or an unpaired Student’s t test was used to determine the statistical significance of intergroup differences (* P<0.05; ** P<0.01; *** P<0.001; **** P<0.0001). Of note, due to the limited sample size, the data do not obey the normal distribution, and the statistical analysis uses a non-parametric test.

## Results

### Clinical and Pathological Characteristics of COVID-19 Patients

We enrolled 23 recovered COVID-19 patients, including 12 mild patients and 11 severe patients in this study. The COVID-19 case definition and clinical classification based on severity were defined according to the New Coronavirus Pneumonia Prevention and Control Protocol for COVID-19 (6th edition) released by the National Health Commission of China. The main symptoms were fever (87.0%, 20/23), fatigue (56.5, 13/23), myalgia (34.8%, 8/23), dyspnea (34.8%, 8/23), dry cough (30.4%, 7/23), expectoration (26.1%, 6/23), chest tightness (26.1%, 6/23), pharyngalgia (13.0%, 3/23), and diarrhea (8.7%, 2/23). On admission, all 23 patients had double lung inflammation on CT scans, and they became normal (17.4%, 4/23), showed significant improvement (52.2%, 12/23) or showed improvement (30.4%, 7/23) when they were out of the hospital. Among the 23 patients, the median age was 56.0 years (40.0-67.0), and the age difference between the mild and severe patients was not significant (54.5 vs. 59.0). Of note, the lymphocyte count, lymphocyte percentage, and albumin level were significantly lower in the severe patients than in the mild patients, and the neutrophil percentage, erythrocyte sedimentation rate (ESR), and C-reactive protein level were significantly higher in the severe patients than in the mild patients. Although the level of hemoglobin and neutrophil count were significantly different between the mild and severe patients, they were still within the normal range. In contrast, the level of IL6 was not significantly different between the mild and severe patients but was higher than the normal range. It is worth noting that in this cohort all 11 severe patients and 3 of 12 mild patients were treated with corticosteroids. The laboratory findings and clinical characteristics of these patients on admission are shown in **Table 1** and **Supplementary Table S1**. All 23 patients recovered and were discharged from the hospital. The median days from symptom onset to discharge for the patients were 33.0 days, and the median days of the severe patients were longer than those of the mild patients (38.0 vs. 30.5).

**Table 1:** Laboratory findings of patients with COVID-19.

	Normal range	Total patients	Mild patients	Severe patients	P
Age, years		56.0 (40.0-67.0)	54.5 (38.8-65.5)	59.0 (40.0-74.0)	0.5353
Time onset to discharge		33.0 (27.0-41.0)	30.5 (25.8-34.8)	38.0 (27.0-41.0)	0.5882
Body temperature		38.1 (36.5-39.5)	37.9 (36.5-39.2)	38.2 (36.5-39.5)	0.3819
White blood cell count, x 10^9^/L	3.7-9.2	5.2 (2.8-10.5)	4.5 (3.0-7.0)	6.1 (2.8-10.5)	0.0637
Neutrophil, x 10^9^/L	2.0-7.0	3.7 (1.6-9.8)	2.6 (1.6-4.4)	5.0 (1.9-9.8)	0.0060**
Neutrophil percentage, %	50-70	67.6 (38.1-94.1)	56.1 (38.1-76.0)	80.1 (62.0-94.1)	<0.0001****
Lymphocyte, x 10^9^/L	0.8-4.0	1.1 (0.4-2.2)	1.5 (0.8-2.2)	0.7 (0.4-1.2)	<0.0001****
Lymphocyte percentage, %	20-40	25.2 (3.3-49.4)	34.8 (16.9-49.4)	14.7 (3.3-25.6)	<0.0001****
Platelet, x 10^9^/L	101-320	192 (131-326)	186 (131-273)	199 (137-326)	0.5386
Hemoglobin	113-151	138 (112-158)	143 (131-158)	132 (112-154)	0.0383*
Albumin, g/L	35-55	38.1 (23.0-50.0)	40.9 (34.0-50.0)	35.0 (23.0-45.0)	0.0237*
ESR, mm/L	0-20	38.1 (2.0-85.0)	21.6 (2.0-63.0)	56.2 (18.0-85.0)	0.0004***
C-reactive protein, mg/L	0.068-8.2	17.2 (0.4-84.9)	5.9 (0.4-21.9)	28.4 (1.0-84.9)	0.0108*
Interleukin-6, pg/mL	0-7	15.5 (1.0-96.1)	9.4 (1.0-26.5)	22.1 (3.2-96.1)	0.1229
Procalcitonin, ng/mL	0-0.05	0.08 (0.02-0.53)	0.043 (0.02-0.09)	0.12 (0.03-0.53)	0.1009

Data are mean (SD) or median (25^th^, 75^th^). *P < 0.05; **P < 0.01; ***P < 0.001; ****P < 0.0001.

To study whether anti-SARS-CoV-2 immune responses persisted for a long time in recovered COVID-19 patients, we followed the discharged patients and collected blood samples 1-3 times from 8 to 16 weeks after symptom onset; the blood sampling time of every recovered subject is displayed in **Supplementary Table S1**. To better analyze the dynamic responses over time, we divided the blood samples into 3 groups (8-10, 11-13, and 14-16 weeks after symptom onset), and each group included both mild and severe recovered patients.

### Phenotypic Analysis of PBMCs From Convalescent COVID-19 Patients

**Figure 1:**
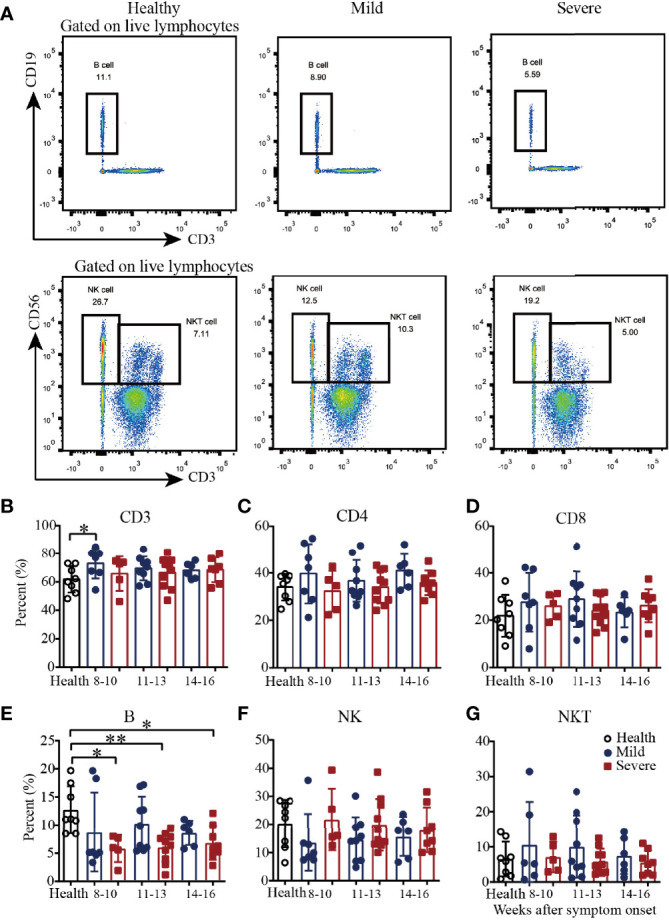
Phenotypic analysis of PBMCs from recovered COVID-19 patients. Representative flow cytometry plots showing the expression patterns of different immune cell subsets in recovered COVID-19 patients **(A)**. Comparison of the frequencies of CD3^+^ **(B)**, CD4^+^ **(C)**, CD8^+^ **(D)**, B cells **(E)**, NK cells **(F)** and NKT cells **(G)** between mild and severe patients from 8 to 16 weeks after symptom onset (Health, n=8; patient, n=22). *P<0.05; **P<0.01.

To explore cellular immune responses, COVID-19 patient PBMCs were isolated from the whole blood and then phenotypically analyzed by flow cytometry (**Figure 1A** and **Supplementary Figure 1**). Compared with those in healthy subjects, the percentages of CD3^+^ T cells in the mild patients were significantly higher during weeks 8-10, and then became similar during weeks 11-13 and 14-16 (**Figure 1B**). For the percentages of CD4^+^ and CD8^+^ T cells, there were no significant difference among the mild patients, severe patients and healthy controls from 8-10 to 14-16 weeks (**Figures 1C, D**). Of note, the percentages of B cells were significantly lower in the severe patients from 8-10 to 14-16 weeks but not in the mild patients (**Figure 1E**). The percentages of natural killer (NK) cells and natural killer T (NKT) cells were similar among the healthy subjects and the mild and severe patients (**Figures 1F, G**). Interestingly, from 8-10 to 14-16 weeks, there was a slight trend toward increased frequencies of NK cells in the mild patients, which was consistent with our previous findings (**Figure 1F**). Additionally, from 8-10 to 14-16 weeks, there was a slight trend toward a decreased frequency of NKT cells, which was similar to the frequency of the healthy controls (**Figure 1G**).

### Macrophage Analysis of PBMCs From Convalescent COVID-19 Patients

**Figure 2:**
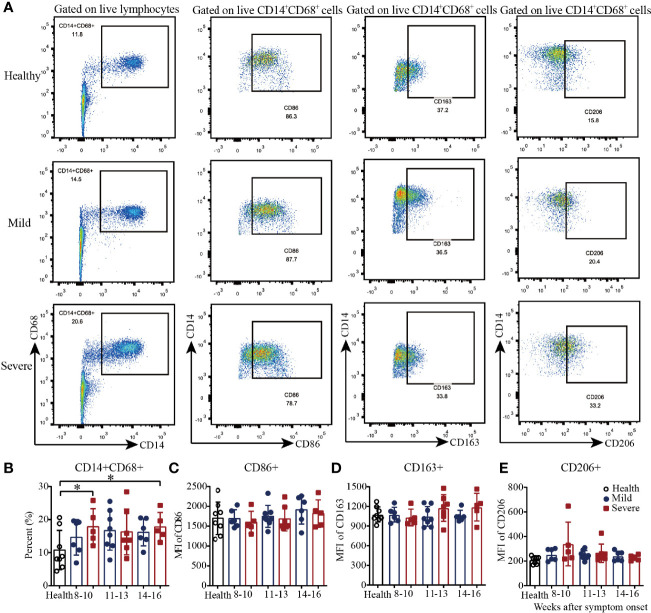
Phenotypic analysis of macrophages in PBMCs from recovered COVID-19 patients. Representative flow cytometry plots showing the expression patterns of different macrophage subsets in recovered COVID-19 patients **(A)**. Comparison of the frequencies of CD14^+^CD68^+^ **(B)**, CD86^+^ **(C)**, CD163^+^ **(D)**, and CD206^+^ **(E)** macrophages between the mild and severe patients from 8 to 16 weeks after symptom onset (Health, n=8; patient, n=22). *P<0.05.

Then, we analyzed the macrophages in PBMCs from convalescent COVID-19 patients by flow cytometry (**Figure 2A** and **Supplementary Figure 2**). As shown in **Figure 2B**, there was a trend toward increased frequency of CD14^+^CD68^+^ T cells in the mild patients from 8-10 to 11-13 weeks and maintenance during weeks 14-16. Additionally, compared with those in the healthy controls, the percentages of CD14^+^CD68^+^ T cells in the severe patients were significantly higher during both 8-10 weeks and 14-16 weeks; the frequency was also higher but not significantly different during weeks 11-13 (**Figure 2B**). For the absolute quantification of CD86^+^ T cells, there was a slight trend toward an increase from 8-10 to 14-16 weeks in both the mild and severe patients (**Figure 2C**). From 8-10 to 14-16 weeks, the absolute quantification of CD163^+^ T cells in the severe patients slightly increased, while it remained at the same level in the mild patients, which was similar to that in the healthy controls (**Figure 2D**). Interestingly, similar to the results for CD163^+^ cells, the absolute quantification of CD206^+^ cells remained at a similar level in the mild patients, which was similar to that in the healthy controls, while it decreased in the severe patients from 8-10 to 14-16 weeks (**Figure 2E**). These data suggested that the dynamics of macrophages with different phenotypes varied during recovery in COVID-19 patients.

### Memory T Cell Subsets of PBMCs From Convalescent COVID-19 Patients

**Figure 3:**
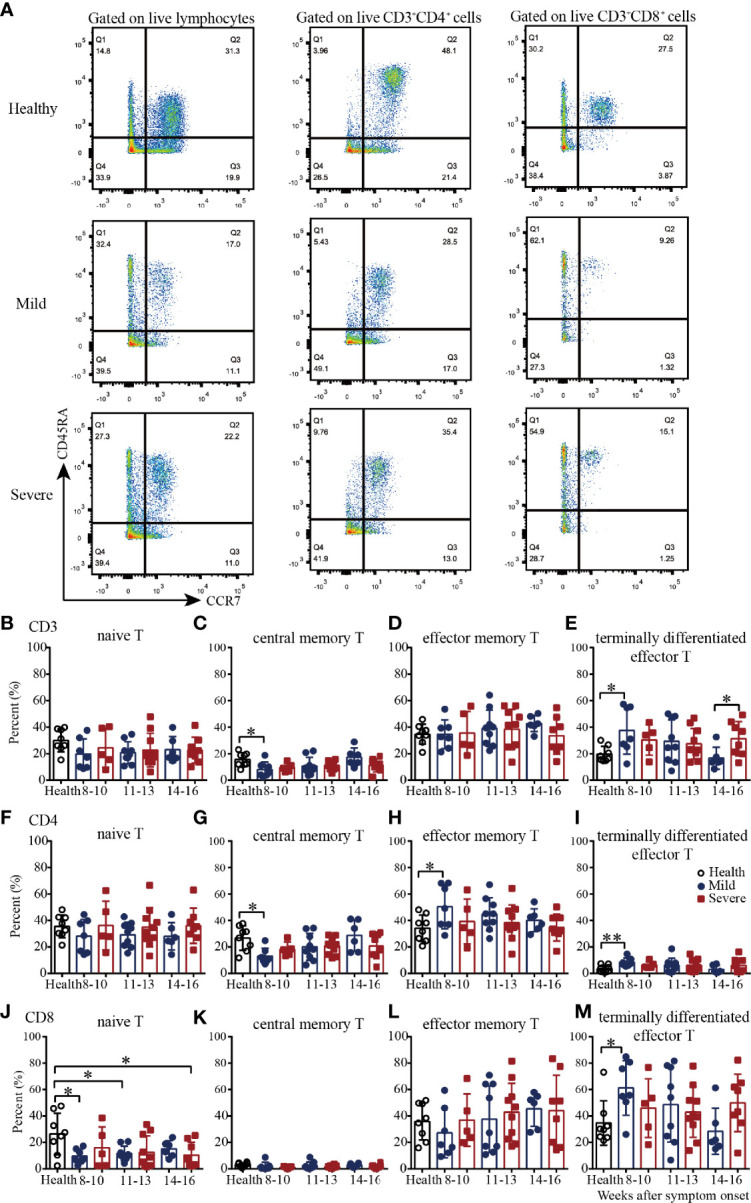
T cell subsets of PBMCs from recovered COVID-19 patients. Representative flow cytometry plots showing the expression patterns of different naïve/memory T-cell subsets in recovered COVID-19 patients **(A)**. Comparison of the frequencies of naïve **(B)**, central memory **(C)**, effector memory **(D)**, and terminally differentiated effector **(E)** CD3^+^ T cells between the mild and severe patients from 8 to 16 weeks after symptom onset. Comparison of the frequencies of naïve **(F)**, central memory **(G)**, effector memory **(H)**, and terminally differentiated effector **(I)** CD4^+^ T cells between the mild and severe patients from 8 to 16 weeks after symptom onset. Comparison of the frequencies of naïve **(G)**, central memory **(K)**, effector memory **(L)**, and terminally differentiated effector **(M)** CD8^+^ T cells between the mild and severe patients from 8 to 16 weeks after symptom onset (Health, n=8; patient, n=22). *P<0.05; **P<0.01.

We next examined four well-defined memory T cell subsets, including naïve, central memory, effector memory and terminally differentiated effector T cells by flow cytometry in convalescent COVID-19 patients (**Figure 3A** and **Supplementary Figure 3**). In the healthy subjects, the CD4^+^:CD8^+^ cell ratios of naïve, central memory, effector memory and terminally differentiated effector T cells were 1.3, 8.5, 0.95 and 0.1, respectively, which indicated that the majority of central memory T cells were CD4^+^ cells, the majority of terminally differentiated effector T cells were CD8^+^ cells, and the effector memory T cells were evenly divided between CD4^+^ and CD8^+^ cells (**Figures 3B–M** and **Supplementary Figure 4**). In the recovered patients, the CD4^+^:CD8^+^ cell ratios of naïve, central memory, effector memory and terminally differentiated effector T cells were 2.6, 10.1, 1.1 and 0.1, respectively. Overall, for T cell subsets, the proportional composition of the four cell types in recovered patients was similar to those of the healthy controls (**Figures 3B–M** and **Supplementary Figure 4**).

Then we evaluated the four T cell subsets according to the disease severity. Compared with those in the healthy controls, from 8-10 to 14-16 weeks, the percentages of naïve CD8^+^ T cells in the mild and severe patients were lower; the percentages were significantly lower in the mild patients during weeks 8-10 and 11-13 and in the severe patients during weeks 14-16 (**Figure 3J**). The percentages of central memory CD3^+^ and CD4^+^ T cells in the mild patients were increased from 8-10 to 14-16 weeks; they were significantly lower than those in the healthy controls during weeks 8-10 but reached similar levels during weeks 14-16 (**Figures 3C, G**). Interestingly, the percentages of effector memory CD4^+^ T cells in the mild patients were decreased from 8-10 to 14-16 weeks; these percentages were significantly higher than those in the healthy controls during weeks 8-10 but reached similar levels during weeks 14-16 (**Figure 3H**). For terminally differentiated effector T cells, the percentages of CD3^+^, CD4^+^ and CD8^+^ cells in the mild patients were all decreased from 8-10 to 14-16 weeks; these percentages were significantly higher than those in the healthy controls during weeks 8-10 but reached similar levels during weeks 14-16 (**Figures 3E, I, M**). Of note, there were no significant differences in the percentages of the four cell types between the healthy controls and severe patients, or between the mild and severe patients (**Figures 3B–M**).

### T-Cell Responses to SARS-CoV-2 in Recovered COVID-19 Patients

**Figure 4:**
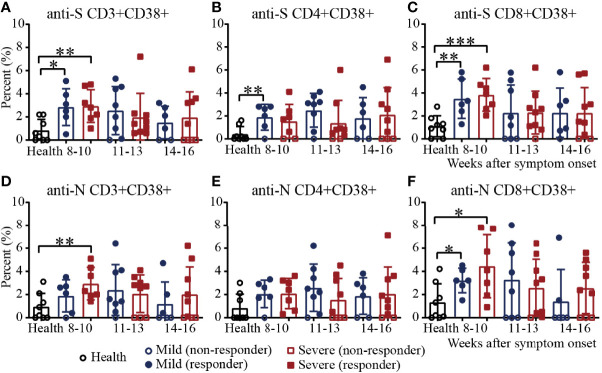
Activation of T cells against the SARS-CoV-2 S/N protein peptide pool in recovered COVID-19 patients. Comparison of the CD3^+^CD38^+^ **(A)**, CD4^+^CD38^+^ **(B)** or CD8^+^CD38^+^ **(C)** S protein peptide pool or the CD3^+^CD38^+^ **(D)**, CD4^+^CD38^+^ **(E)** or CD8^+^CD38^+^ **(F)** N protein peptide pool between the mild and severe patients from 8 to 16 weeks after symptom onset (Health, n=8; patient, n=22). *P<0.05; **P<0.01; ***P<0.001.

To further assess virus-specific cellular immunity, PBMCs were treated with the SARS-CoV-2 S antigen peptide pool and N antigen pool (23–28), and cytokine production were measured by flow cytometry. Considering the background of peptide stimulation, we subtracted the DMSO-stimulated response from the peptide-stimulated PBMCs of COVID-19 patients or healthy controls. First, we evaluated the activation marker of SARS-CoV-2 S/N-specific T cells in recovered COVID-19 patients (29) (**Supplementary Figure 5**). For S-specific T cells, during weeks 8-10, the percentages of CD3^+^CD38^+^ T cells in mild and severe patients were significantly higher than those in the healthy controls. The proportion of patients positive for CD3^+^CD38^+^ T cells was 100% during weeks 8-10, while the number of negative samples for CD3^+^CD38^+^ T cells increased gradually during the following 11-13 and 14-16 weeks (**Figure 4A**). For S-specific CD4^+^CD38^+^ T cells, the percentage in mild patients during 8-10 was significantly higher than that in the healthy controls (**Figure 4B**). For S-specific CD8^+^CD38^+^ T cells, the results were similar with S-specific CD3^+^CD38^+^ T cells (**Figure 4C**). For N-specific CD3^+^CD38^+^ T cells, during weeks 8-10, the percentage in severe patients was significantly higher than that in the healthy controls, and only one mild patient was negative for N-specific CD3^+^CD38^+^ T cells (**Figure 4D**). For N-specific CD4^+^CD38^+^ T cells, there was no significant difference between the patients and healthy controls (**Figure 4E**). For N-specific CD8^+^CD38^+^ T cells, during weeks 8-10, the percentages in mild and severe patients were significantly higher than those in the healthy controls. The proportion of patients positive for N-specific CD8^+^CD38^+^ T cells was 100% during weeks 8-10, while the number of negative sample increased gradually during the following 11-13 and 14-16 weeks (**Figure 4F**). Of note, the percentages of S- and N- specific CD8^+^CD38^+^ T cells were both significantly higher than those of the CD4^+^CD38^+^ T cells in recovered patients (**Figures 4B, C, E, F**). In addition, the percentages of S-specific CD38^+^ T cells was similar to that of N-specific CD38^+^ T cells (**Figures 4A, D**).

**Figure 5:**
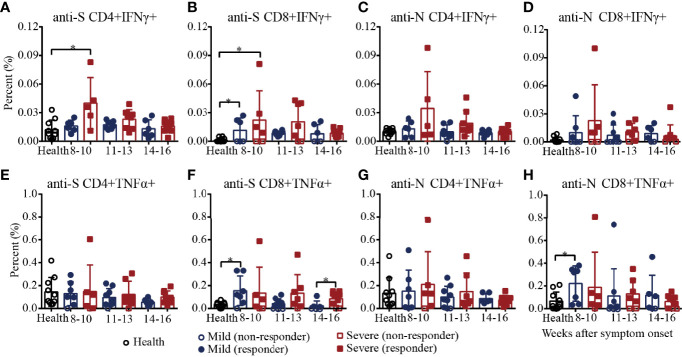
T-cell responses to SARS-CoV-2 in recovered COVID-19 patients measured by flow cytometric analysis. Comparison of the IFNγ responses against the CD4^+^ **(A)** or CD8^+^ **(B)** S protein peptide pool or the CD4^+^ **(C)** or CD8^+^ **(D)** N protein peptide pool between the mild and severe patients. Comparison of the TNFα responses against the CD4^+^ **(E)** or CD8^+^ **(F)** S protein peptide pool or the CD4^+^ **(G)** or CD8^+^ **(H)** N protein peptide pool between the mild and severe patients. *P<0.05.

Then, we characterized the cytokine expression of SARS-CoV-2 S/N-specific T cells in recovered COVID-19 patients (**Supplementary Figure 6** and **Supplementary Figure 7**). As shown in **Figure 5A**, the percentages of SARS-CoV-2 S-specific IFNγ^+^CD4^+^ T cells in the severe patients decreased from 8-10 to 14-16 weeks and were significantly higher than those in the healthy controls during weeks 8-10, and the percentages of S-specific IFNγ^+^CD4^+^ T cells in the mild patients were similar to those in the healthy controls. For S-specific IFNγ^+^CD8^+^ T cells, the percentages in the mild and severe patients were both significantly higher than those in the healthy controls during weeks 8-10, and there was no significant difference between the mild and severe patients (**Figure 5B**). For N-specific IFNγ^+^ CD4^+^ and CD8^+^ T cells, the percentages in the mild and severe patients were both similar to those in the healthy controls (**Figures 5C, D**). For S/N-specific-TNFα^+^CD4^+^ T cells, there was no difference among the mild, severe and healthy controls (**Figure 5E, G**). For S-specific TNFα^+^CD8^+^ T cells, the percentage in the mild patients was statistically higher than that in the healthy controls during weeks 8-10, which was similar to that in the severe patients. During weeks 14-16, the percentages in the severe patients were significantly higher than those in the mild patients because the percentages in the mild patients declined from weeks 8-10 to 14-16, while the percentages in the severe patients were maintained until weeks 14-16 (**Figure 5F**). Consistent with the results for S-specific TNFα^+^CD8^+^ T cells, the percentage of N-specific TNFα^+^CD8^+^ T cells in the mild patients was also significantly higher than that in the healthy control during weeks 8-10, which was similar to that in the severe patients (**Figure 5H**).

**Figure 6:**
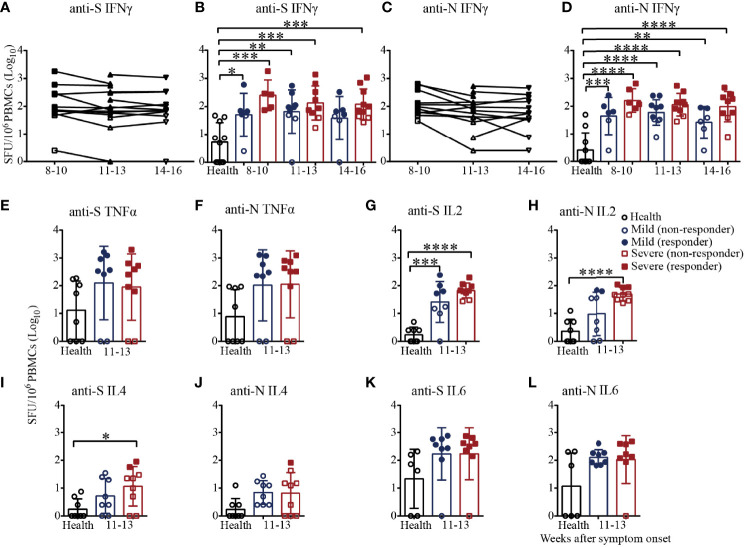
T-cell responses to SARS-CoV-2 in recovered COVID-19 patients measured by ELISpot analysis. IFNγ ELISpot analysis of responses against the S **(A)** or N **(C)** protein peptide pool in COVID-19 patients from 8 to 16 weeks after symptom onset. Comparison of the IFNγ responses against the S **(B)** or N **(D)** protein peptide pool between mild and severe patients from 8 to 16 weeks after symptom onset. Comparison of the TNFα responses against the S **(E)** or N protein peptide pool **(F)** between mild and severe patients from 11 to 13 weeks. Comparison of the IL2 responses against the S **(G)** or N **(H)** protein peptide pool between mild and severe patients from 11 to 13 weeks. Comparison of the IL4 responses against the S **(I)** or N **(J)** protein peptide pool between mild and severe patients from 11 to 13 weeks. Comparison of the IL6 responses against the S **(K)** or N **(L)** protein peptide pool between mild and severe patients from 11 to 13 weeks (Health, n=10; patient, n=22). *P<0.05; **P<0.01; ***P<0.001; ****P<0.0001.

Finally, we adopted the ELISpot assay, a more sensitive method, to evaluate the virus-specific cellular immunity mediated by PBMCs. As shown in **Figures 6A, B**, the numbers of IFNγ-secreting S-specific T cells remained at a high mean level of 204.1 until 14-16 weeks after symptom onset, although the number decreased over time, indicating that the recovered patients developed SARS-CoV-2 S-specific T-cell responses that lasted for a long time. Moreover, the numbers of IFNγ-secreting S-specific T cells were maintained at a similar level in the mild patients in the different time periods but slightly gradually decreased in the severe patients from 8-10 to 14-16 weeks after symptom onset. Compared to the healthy subjects, the numbers of IFNγ-secreting S-specific T cells in mild and severe patients were significantly higher from 8-10 to 14-16 weeks. Although the numbers of IFNγ-secreting S-specific T cells in the severe patients were higher than those in the mild patients at every time point, there were no significant differences. In addition, the IFNγ-secreting N-specific T cells in the recovered patients were similar to but slightly lower than the S-specific T cells (**Figures 6C, D**). Of note, the proportion of patients positive for IFNγ-secreting S-specific T cells was maintained at 71.4% to 81.8%, and the proportion of the severe patients was higher than that of the mild patients (8-10 weeks, 100% vs. 66.7%; 11-13 weeks, 77.8% vs. 66.7%; 14-16 weeks, 70% vs. 57.1%). For IFNγ-secreting N-specific T cells, the proportion of positive patients declined from 81.8% to 58.3%. From 8-10 to 14-16 weeks, the proportion of positive severe patients was maintained at approximately 80%, while the proportion of positive mild patients declined from 66.7% to 28.6%, indicating that IFNγ-secreting SARS-CoV-2-specific T cells gradually disappear in mild COVID-19 patients (**Figures 6B, D**).

We also measured the numbers of TNFα, IL2, IL4 and IL6-secreting S/N-specific T cells in the recovered patients during 11-13 weeks after symptom onset (**Figures 6E–L**). As shown in **Figure 6E**, TNFα-secreting S-specific T cells were present in 75% (6/8) of the mild recovered patients and 77.8% (7/9) of the severe recovered patients, which was consistent with the results for N-specific T cells (**Figure 6F**), suggesting that the level of TNFα responses against the S antigen pool was similar to that against the N antigen pool. In terms of IL2 secretion, the proportions of patients with positive S-specific T cells in the mild and severe patients were 50% (4/8) and 77.8% (7/9), respectively, while the proportions were only 25% (2/8) and 33.3% (3/9) for N-specific T cells, respectively. For IL2-secreting T cells, the number of S-specific T cells in the mild and severe patients and the number of N-specific T cells in the severe patients were significantly higher than those of the health subjects (**Figures 6G, H**). Of note, all the mild patients were negative for IL4-secreting S/N-specific T cells, while the positive proportions were 22.2% (2/9) and 11.1% (1/9) in the severe patients (**Figures 6I, J**). The proportion of patients positive for IL6-secreting S-specific T cells was 87.5% (7/8) for both the mild and severe patients, while the proportions were 100% (8/8) and 87.5%, respectively, for the N antigen pool (**Figures 6K, L**). The data suggested that IFNγ, TNFα, IL2 and IL6 might be the main cytokines involved in the T-cell immunity in recovered patients. Additionally, it was worth noting that the secreted IFNγ and IL2 level in severe patients was higher than that in mild patients.

## Discussion

We established a study cohort of recovered COVID-19 patients to investigate the longitudinal dynamics of adaptive immunity and SARS-CoV-2-specific cellular immunity from 8-10 to 14-16 weeks after symptom onset. This study had several important highlights. (i) During weeks 8-10, the percentage of activation of SARS-CoV-2 S/N-specific T cells in recovered patients was significantly higher than that in healthy subjects. And the activation percentage of S/N-specific CD8^+^ T cells in recovered patients was significantly higher than that of CD4^+^ T cells. (ii) SARS-CoV-2 specific T-cell responses were strongly biased toward the expression of Th1 cytokines, included the cytokines IFNγ, TNFα and IL2. (iii) The number of IFNγ-secreting S-specific T cells in recovered patients were slightly higher than that of N-specific T cells. (iv) The secreted IFNγ and IL2 level in severe patients was higher than that in mild patients. Overall, the SARS-CoV-2 S/N-specific T-cell responses in recovered patients were strong, and virus-specific immunity was present until 14-16 weeks after symptom onset.

In this study, T-cell responses against the SARS-CoV-2 S or N antigen could be detected in majority of the recovered COVID-19 patients and persisted until 4 months after symptom onset. The virus-specific T-cell responses were strongly biased toward the expression of Th1 cytokines (IFNγ, TNFα and IL2) with minimal expression of Th2 cytokines (IL4 and IL6), which was consistent with previous studies (14, 30). Ni et al (31) found that although the numbers of IFNγ-secreting S-RBD-specific T cells in the mild patients were much lower than those of NP-specific T cells, they could be detected in more patients than T cells specific for other viral proteins. In this cohort, we found that the percentages of patients positive for IFNγ-secreting S-specific T cells were higher than those positive for N-specific T cells, and so was the number of IFNγ-secreting T cells. This discrepancy may be due to the different antigenic stimuli used or individual differences among the patients. Of note, the cellular immune response in severe patients was stronger than that in mild patients, which was consistently with other studies (12, 32).

This study had several limitations. First, the study cohort was relatively small size, and a larger cohort is needed to fully identify the characteristics of cellular immunity in COVID-19 patients. Second, we restricted this study cohort to individuals who recovered from mildly or severely symptomatic COVID-19. Thus, we did not characterize cellular immunity in asymptomatic individuals. Moreover, we followed the discharged patients only from 8-10 to 14-16 weeks after symptom onset, and we need to follow a cohort of individuals from symptom onset to discharge and follow-up for 1 year or longer. Additionally, owing to the lack of detailed information on common cold history or matched blood samples collected prior to exposure to SARS-CoV-2, we could not confirm whether any cross-reactive immunity existed in this study cohort. Finally, 10 healthy donors with a median age of 31.0 years, which are not age-matched with recovered patients with a median age of 56.0 years.

In summary, we characterized the longitudinal dynamics of cellular immunity in recovered COVID-19 patients. Using multiple experimental methods, T-cell responses against the SARS-CoV-2 S or N antigen could be detected in all recovered COVID-19 patients and persisted until 4 months after symptom onset, indicating that the T-cell memory is likely to persist long-term in COVID-19 patients.

## Data Availability Statement

The original contributions presented in the study are included in the article/**Supplementary Material**. Further inquiries can be directed to the corresponding authors.

## Ethics Statement

The studies involving human participants were reviewed and approved by the Fifth Medical Center of Chinese PLA General Hospital (2020031D). The patients/participants provided their written informed consent to participate in this study.

## Author Contributions

C-FQ, C-QB, MC, YC, and FL contributed to the study design, data analysis and interpretation. C-FQ, MC, CZ, J-JH, QM, GW, and HZ contributed to writing and editing the manuscript. HL, JZ, Y-HQ, NY, YZ, and C-QB contributed to collecting clinical specimens. MC, CZ, ZZ, and R-TL contributed to performing all the experiments. All authors contributed to the article and approved the submitted version.

## Funding

This work was supported by the National Key Research and Development Project of China (2020YFC0842200, and 2020YFA0707801), the Special Grant from AMS (JK2020NC002), and the National Natural Science Foundation (No. 82041044). C-FQ was supported by the National Science Fund for Distinguished Young Scholar (No. 81925025), and the Innovative Research Group (No. 81621005) from the NSFC, and the Innovation Fund for Medical Sciences (No.2019-I2M-5-049) from the Chinese Academy of Medical Sciences.

## Conflict of Interest

The authors declare that the research was conducted in the absence of any commercial or financial relationships that could be construed as a potential conflict of interest.

## Publisher’s Note

All claims expressed in this article are solely those of the authors and do not necessarily represent those of their affiliated organizations, or those of the publisher, the editors and the reviewers. Any product that may be evaluated in this article, or claim that may be made by its manufacturer, is not guaranteed or endorsed by the publisher.
