# Supplementary material for: Longitudinal Dynamics of Cellular Responses in Recovered COVID-19 Patients
Source: Front Immunol. 2022 May 19;13:911859. doi: 10.3389/fimmu.2022.911859 (PMC9161166; doi:10.3389/fimmu.2022.911859)
Supplement: Supplementary file 1 [file DataSheet_1.docx]

Supplementary Material

## Supplementary Figures

##
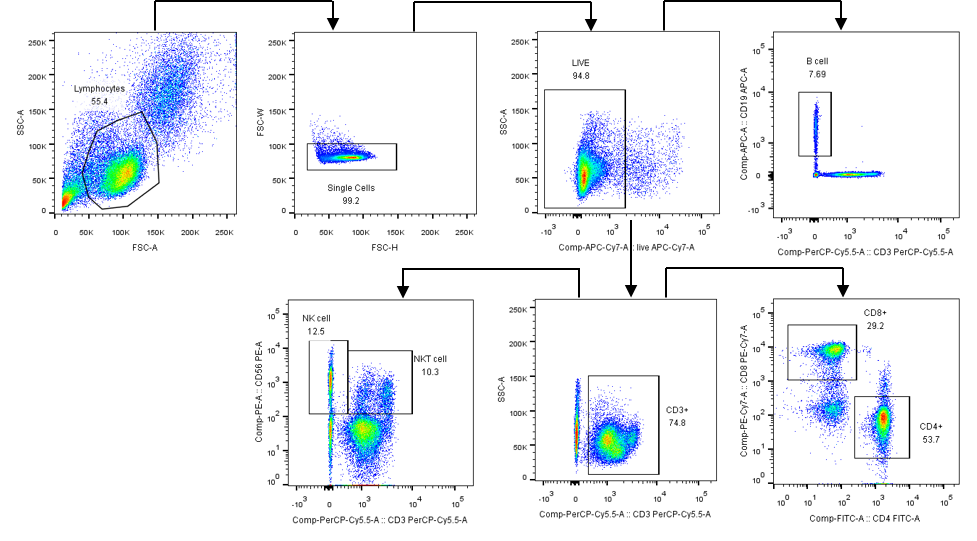


## Supplementary Figure 1. Gating strategy for phenotypic analysis of PBMCs from convalescent COVID-19 patients.

**
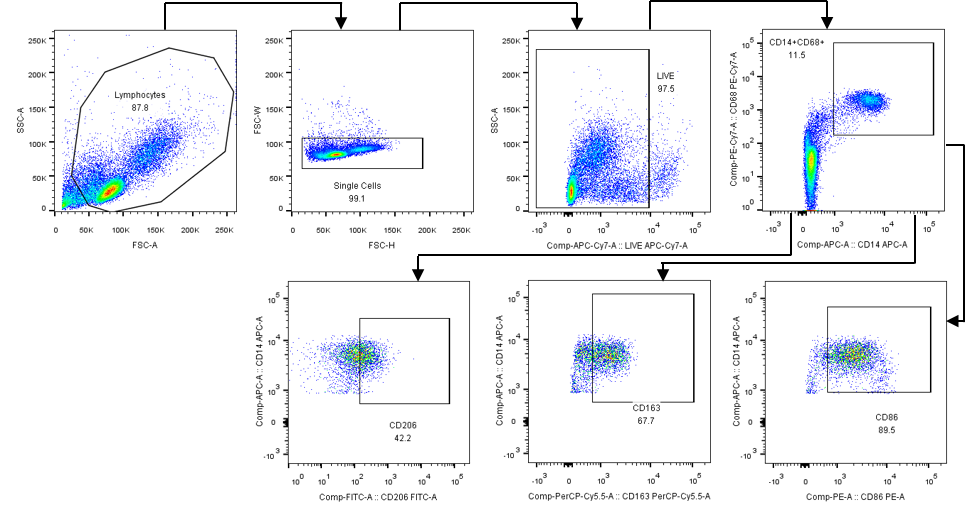
**

**Supplementary Figure 2**. Gating strategy for macrophage analysis of PBMCs from convalescent COVID-19 patients.

**
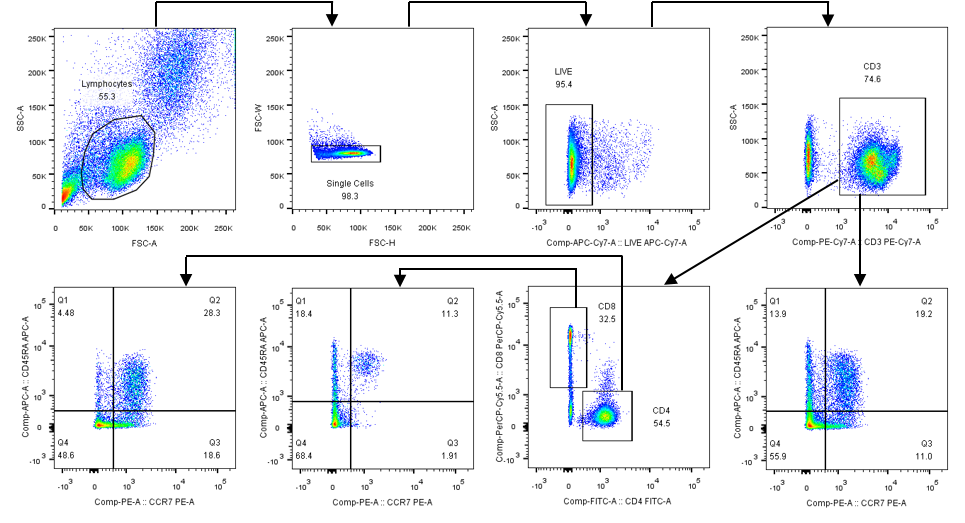
**

**Supplementary Figure 3**. Gating strategy for T cell subsets of PBMCs from convalescent COVID-19 patients.

**
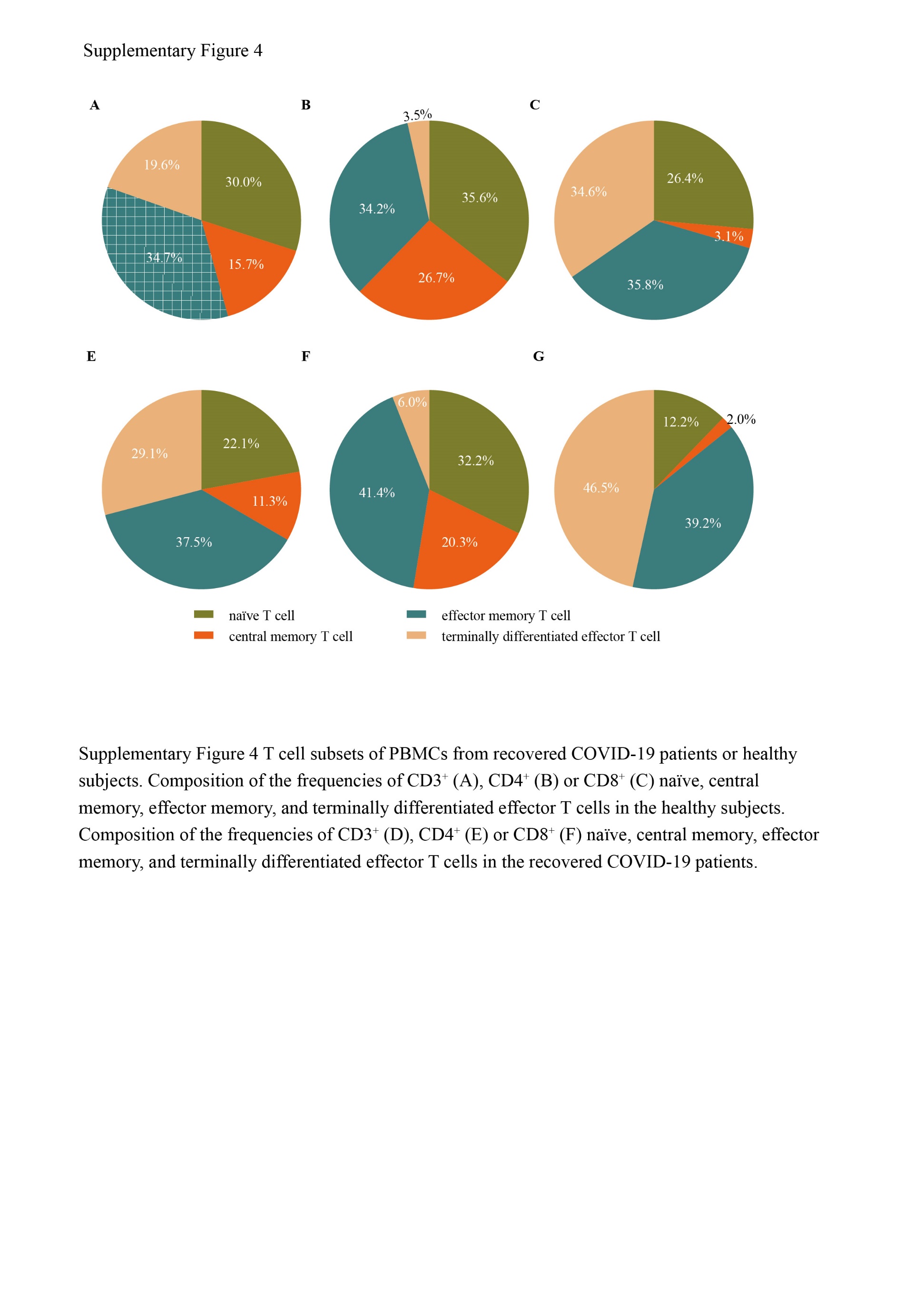
**

**Supplementary Figure 4**. T cell subsets of PBMCs from recovered COVID-19 patients or healthy subjects. Composition of the frequencies of CD3+ **(A)**, CD4+ **(B)** or CD8+ **(C)** naïve, central memory, effector memory, and terminally differentiated effector T cells in healthy subjects. Composition of the frequencies of CD3+ **(D)**, CD4+ **(E)** or CD8+ **(F)** naïve, central memory, effector memory, and terminally differentiated effector T cells in recovered COVID-19 patients.

**
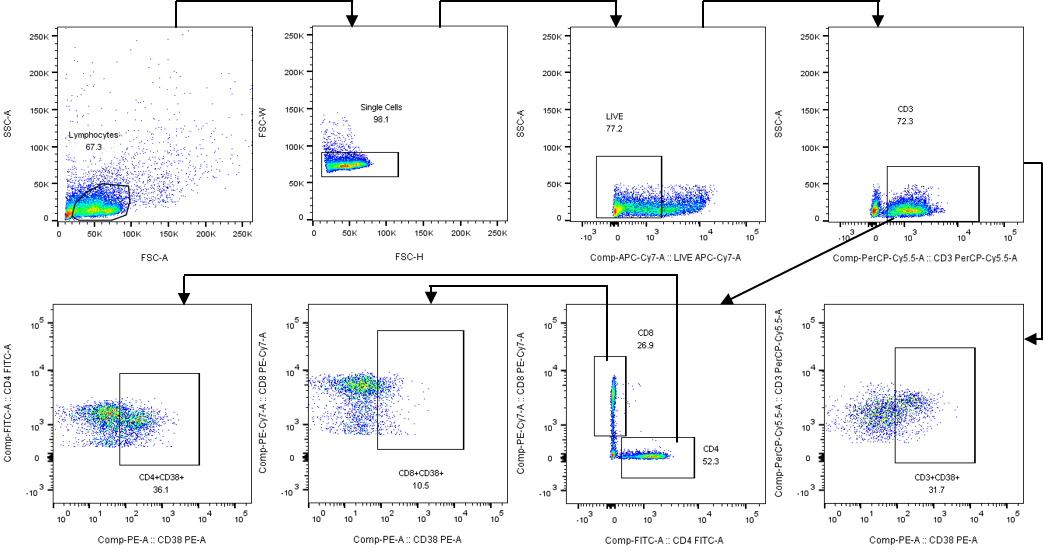
**

**Supplementary Figure 5**. Gating strategy for CD38^+^ T cells of PBMCs from convalescent COVID-19 patients.

**
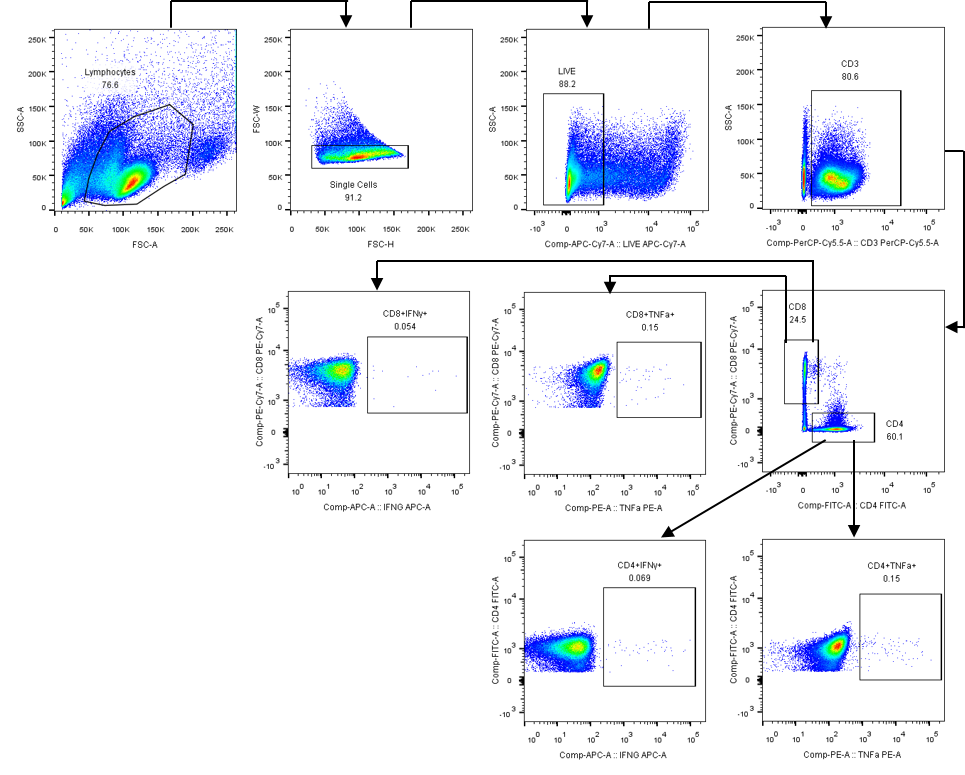
**

**Supplementary Figure 6**. Gating strategy for IFNγ^+^ or TNFα^+^ T cells of PBMCs from convalescent COVID-19 patients.

**
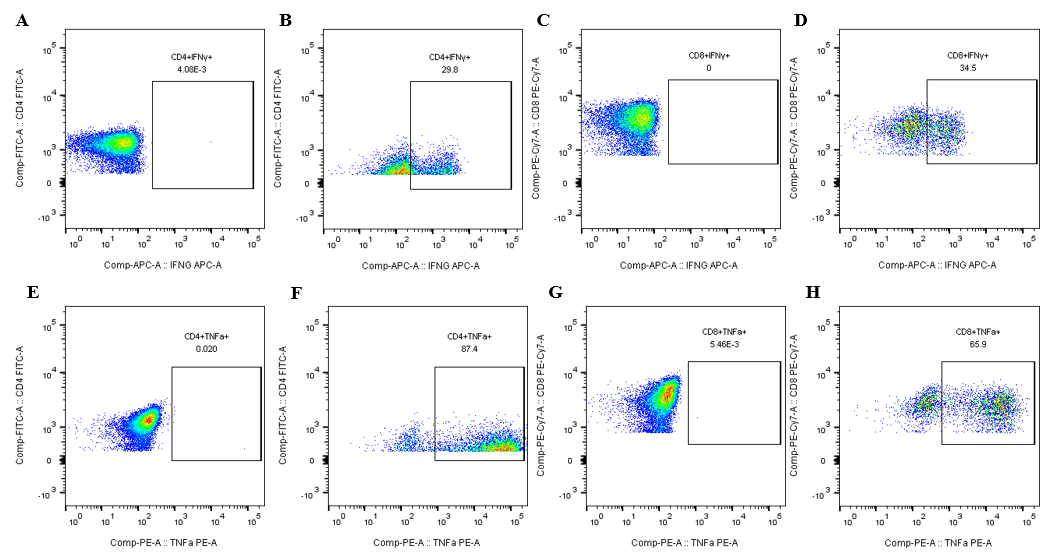
**

**Supplementary Figure 7**. Negative and positive control for IFNγ^+^ or TNFα^+^ T cells of PBMCs from convalescent COVID-19 patients or healthy subjects. PBMCs were stimulated with DMSO **(A)** or PMA **(B)**, respectively, as the negative or positive control for CD4^+^IFNγ^+^ T cell analysis. PBMCs were stimulated with DMSO **(C)** or PMA **(D)**, respectively, as the negative or positive control for CD8^+^IFNγ^+^ T cell analysis. PBMCs were stimulated with DMSO **(E)** or PMA **(F)**, respectively, as the negative or positive control for CD4^+^TNFα^+^ T cell analysis. PBMCs were stimulated with DMSO **(G)** or PMA **(H)**, respectively, as the negative or positive control for CD8^+^TNFα^+^ T cell analysis.
