# Supplementary material for: Longitudinal Dynamics of Cellular Responses in Recovered COVID-19 Patients
Source: Front Immunol. 2022 May 19;13:911859. doi: 10.3389/fimmu.2022.911859 (PMC9161166; doi:10.3389/fimmu.2022.911859)
Supplement: Supplementary file 2 [file Table_1.docx]

**Supplementary Table S1** Clinical and pathological characteristics of the COVID-19 patients

| NO. | Sex | Age | Diagnosis | Lymphocyte count (x10^9^/L) | Main symptom | Days in  hospital | CS  therapy | BT  CT scan | Discharge  CT scan | Sampling  time |
| --- | --- | --- | --- | --- | --- | --- | --- | --- | --- | --- |
| #1 | M | 36 | severe | 0.7 | fever, expectoration, polypnea | 15 | Yes | DLB | improvement | 11,15 |
| #2 | M | 37 | mild | 2.24 | fever, dry cough, pharyngalgia, fatigue, myalgia | 18 | Yes | DLB | significant improvement | 8,12 |
| #3 | M | 64 | mild | 1.75 | myalgia | 19 | No | DLB | normal | 8,10 |
| #4 | M | 66 | mild | 1.39 | asymptomatic | 15 | No | DLB | improvement | 8,13 |
| #5 | F | 72 | mild | 1.38 | fever, expectoration, myalgia, chest tightness | 46 | No | DLB | normal | 10,12 |
| #6 | M | 37 | mild | 1.45 | fever, fatigue, diarrhea | 32 | No | DLB | normal | 8,11,15 |
| #7 | M | 48 | severe | 1.17 | fever, fatigue, myalgia | 26 | Yes | DLB | improvement | 10,15 |
| #8 | F | 53 | mild | 1.16 | fever, dry cough, fatigue, | 27 | Yes | DLB | significant improvement | 9,12,16 |
| #9 | F | 74 | severe | 0.91 | fever, pharyngalgia, fatigue, dyspnea | 33 | Yes | DLB | significant improvement | 8,11,15 |
| #10 | M | 78 | critical | 0.41 | fever, dry cough, fatigue, dyspnea | 32 | Yes | DLB | improvement | 8,11,15 |
| #11 | M | 59 | severe | 1.2 | fever | 23 | Yes | DLB | normal | 9,12 |
| #12 | F | 50 | critical | 0.48 | fever, expectoration, fatigue, myalgia, dyspnea | 20 | Yes | DLB | significant improvement | 8,11,16 |
| #13 | M | 35 | critical | 0.95 | fever, dry cough, fatigue, chest tightness, dyspnea | 17 | Yes | DLB | significant improvement | 8,11 |
| NO. | Sex | Age | Diagnosis | Lymphocyte count (x10^9^/L) | main symptom | Days in  hospital | CS | BT  CT scan | discharge  CT scan | Sampling |
|  |  |  |  |  |  |  | therapy |  |  | time |
| #14 | M | 75 | critical | 0.35 | fever, dry cough, fatigue, chest tightness, dyspnea | 37 | Yes | DLB | improvement | 11,16 |
| #15 | M | 40 | severe | 0.73 | fever, expectoration, pharyngalgia, fatigue, myalgia, chest tightness, dyspnea | 48 | Yes | DLB | significant improvement | 13,16 |
| #16 | M | 44 | mild | 2.02 | fever, dry cough | 27 | No | DLB | significant improvement | 12 |
| #17 | M | 32 | mild | 1.31 | fever, myalgia | 28 | Yes | DLB | significant improvement | 12 |
| #18 | F | 67 | critical | 0.36 | fever, expectoration, fatigue, chest tightness, dyspnea | 22 | Yes | DLB | significant improvement | 11,16 |
| #19 | F | 44 | mild | 1.11 | fever | 20 | No | DLB | significant improvement | 12,16 |
| #20 | M | 58 | mild | 2.01 | fever | 54 | No | DLB | improvement | 13 |
| #21 | F | 56 | mild | 1.53 | fever, fatigue, myalgia | 20 | No | DLB | significant improvement | 15 |
| #22 | F | 60 | severe | 1.68 | fever, expectoration, chest tightness, dyspnea | 32 | Yes | DLB | improvement | 11 |
| #23 | F | 69 | mild | 0.83 | fever, dry cough, fatigue, | 20 | No | DLB | significant improvement | 13 |

Notes: M, male; F, female; BT, before treatment; CS, corticosteroids; DLB, double lung inflammation; Sampling time, weeks after symptom onset

**Supplementary Table S2** SARS-CoV-2 T cell epitopes used in this study

| Sequence | Protein | Mapped (Started-End) |
| --- | --- | --- |
| IRGWIFGTTLDSKTQSLL | S | 101-118 |
| QPFLMDLEGKQGN | S | 173-185 |
| TRFQTLLALHRSYLTPGDSSSGW | S | 236-258 |
| SASFSTFKCYGVSPTKL | S | 371-387 |
| KLPDDFTGCV | S | 424-433 |
| NLDSKVGGNYNYLYRLFR | S | 440-457 |
| YLYRLFRKSNLKPFERDI | S | 451-468 |
| KPFERDISTEIYQ | S | 462-474 |
| VKQIYKTPPIKDFGGFNF | S | 785-802 |
| DSLSSTASALGKLQDVV | S | 936-952 |
| ALNTLVKQL | S | 958-966 |
| LITGRLQSL | S | 996-1004 |
| QLIRAAEIRASANLAATK | S | 1011-1028 |
| RLNEVAKNL | S | 1185-1193 |
| ALNTPKDHI | N | 138-146 |
| LQLPQGTTL | N | 159-167 |
| LLLDRLNQL | N | 222-230 |
| RLNQLESKM | N | 226-234 |
| GMSRIGMEV | N | 316-324 |
| MEVTPSGTWL | N | 322-331 |
